# A Combined Geometric Morphometric and Discrete Element Modeling Approach for Hip Cartilage Contact Mechanics

**DOI:** 10.3389/fbioe.2020.00318

**Published:** 2020-04-21

**Authors:** Jan Van Houcke, Emmanuel A. Audenaert, Penny R. Atkins, Andrew E. Anderson

**Affiliations:** ^1^Department of Orthopaedic Surgery and Traumatology, Ghent University Hospital, Ghent, Belgium; ^2^Department of Orthopaedics, University of Utah, Salt Lake City, UT, United States; ^3^Department of Human Structure and Repair, Ghent University, Ghent, Belgium; ^4^Department of Trauma and Orthopaedics, Addenbrooke’s Hospital, Cambridge University Hospitals NHS Foundation Trust, Cambridge, United Kingdom; ^5^Department of Electromechanics, Op3Mech Research Group, University of Antwerp, Antwerp, Belgium; ^6^Department of Biomedical Engineering, University of Utah, Salt Lake City, UT, United States; ^7^Department of Physical Therapy and Athletic Training, University of Utah, Salt Lake City, UT, United States; ^8^Scientific Computing and Imaging Institute, University of Utah, Salt Lake City, UT, United States

**Keywords:** hip joint, contact mechanics, discrete element analysis, finite element analysis, cartilage prediction

## Abstract

Finite element analysis (FEA) provides the current reference standard for numerical simulation of hip cartilage contact mechanics. Unfortunately, the development of subject-specific FEA models is a laborious process. Owed to its simplicity, Discrete Element Analysis (DEA) provides an attractive alternative to FEA. Advancements in computational morphometrics, specifically statistical shape modeling (SSM), provide the opportunity to predict cartilage anatomy without image segmentation, which could be integrated with DEA to provide an efficient platform to predict cartilage contact stresses in large populations. The objective of this study was, first, to validate linear and non-linear DEA against a previously validated FEA model and, second, to present and evaluate the applicability of a novel population-averaged cartilage geometry prediction method against previously used methods to estimate cartilage anatomy. The population-averaged method is based on average cartilage thickness maps and therefore allows for a more accurate and individualized cartilage geometry estimation when combined with SSM. The root mean squared error of the population-averaged cartilage geometry predicted by SSM as compared to the manually segmented cartilage geometry was 0.31 ± 0.08 mm. Identical boundary and loading conditions were applied to the DEA and FEA models. Predicted DEA stress distribution patterns and magnitude of peak stresses were in better agreement with FEA for the novel cartilage anatomy prediction method as compared to commonly used parametric methods based on the estimation of acetabular and femoral head radius. Still, contact stress was overestimated and contact area was underestimated for all cartilage anatomy prediction methods. Linear and non-linear DEA methods differed mainly in peak stress results with the non-linear definition being more sensitive to detection of high peak stresses. In conclusion, DEA in combination with the novel population-averaged cartilage anatomy prediction method provided accurate predictions while offering an efficient platform to conduct population-wide analyses of hip contact mechanics.

## Introduction

Hip osteoarthritis (OA) is a disabling condition with a lifetime risk of 25% (Murphy et al., 2010). Most cases of hip OA are theorized to be the consequence of unfavorable mechanical conditions (Reijman et al., 2005; Ganz et al., 2008). Structural hip deformities, including developmental dysplasia of the hip, acetabular retroversion, and femoroacetabular impingement are recognized etiologies of hip OA (Ganz et al., 2008; Agricola et al., 2013a, b; Hosnijeh et al., 2017). Structural hip deformities are believed to cause deleterious stresses and strains in the cartilage, resulting in mechanical damage and hip OA (Genda et al., 2001; Mavcic et al., 2002). Still, there is a high prevalence of structural hip deformities amongst the asymptomatic population that show no radiographic evidence of joint space narrowing indicative of OA (Anderson et al., 2016). Thus, the relationship between hip pathoanatomy and OA is not well understood. Although cartilage stresses cannot be measured directly, they can be estimated from computational models. Computational techniques that afford prediction of cartilage stress in appropriately-powered studies would improve understanding of the pathogenesis of hip OA. Yet, the development and analysis of these computer models is time-consuming and technically-challenging due to laborious pre-processing and the need for specific domain expertise, which may explain why most modeling studies of the hip have utilized small sample sizes (Henak et al., 2013).

When incorporating subject-specific anatomy, finite element analysis (FEA) can predict cartilage stresses in good agreement with *in vitro* data (Anderson et al., 2008). Most often, bone is segmented from computed tomography (CT) or magnetic resonance images (MRI) using automatic or semi-automatic segmentation techniques. However, segmentation of hip cartilage is time-consuming (Henak et al., 2013). Given the close-fitting geometry, opposing layers of femoral and acetabular cartilage cannot be reliably identified without the use of contrast enhancement (i.e., arthrography) and traction. Still, even when using arthrography and traction, there are several regions where cartilage must be segmented manually (Henak et al., 2013). Some investigators have attempted to circumvent this problem by representing the hip joint as an idealized sphere or as a joint with constant cartilage thickness (Genda et al., 2001; Yoshida et al., 2006). However, such simplified models yield inaccurate predictions of cartilage stress and strain (Anderson A. E. et al., 2010).

Having an efficient computational method for analyzing cartilage stresses would enable studies of larger sample sizes, which could prove clinically useful for pre-operative planning or intra-operative navigation if stresses could be predicted in near real-time. Discrete element analysis (DEA) provides an attractive alternative to FEA since these models can be solved in less than a minute using a desktop computer (Abraham et al., 2013). Conversely, FEA models may require an hour or more of computing time, even when using a computing cluster (Henak et al., 2013). Typically, DEA models assume bones to be rigid structures, and represent cartilage as an array of springs (Li et al., 1997; Volokh et al., 2007; Abraham et al., 2013). In the studies by Li et al. (1997) and Volokh et al. (2006) femoral head geometry and cartilage thickness were assumed constant and spherical. Abraham et al. (2013) improved upon these prior studies by assigning subject-specific cartilage thickness, and showed that DEA models could predict stresses in good agreement with FEA. However, in the study by Anderson et al. (2008) cartilage geometry was available from a previously validated FEA model in which cartilage geometry was segmented from CT images.

The objective of this study was first, to validate linear and non-linear DEA compared with a previously validated FEA model (Anderson et al., 2008). Second, to present a novel population-averaged cartilage geometry prediction method and evaluate the methodology against generic parameterized methods in terms of anatomical accuracy and resulting contact mechanics as benchmarked against a validated FEA model (Harris et al., 2012). Our method to describe cartilage geometry builds on geometric morphometrics to provide patient specific cartilage anatomy based on population-averaged thickness measurements.

## Materials and Methods

The general workflow for this study was: (1) to develop the discrete element models with both linear and non-linear definitions and benchmark them against a validated FEA model, (2) assign cartilage geometry using a novel methodology based on population-averaged thickness maps and compare it with popular cartilage geometry prediction methods, and (3) evaluate the accuracy of cartilage geometry prediction methods and their impact on contact mechanics using DEA.

### Development of the Discrete Element Model

#### Geometric Morphometric Analysis of Hip Anatomy

Skeletal anatomy of the different hip bones was derived using previously described methodology for automated image segmentation and statistical shape modeling (SSM) (Almeida et al., 2016; Audenaert et al., 2019a, b, 2020). The pipeline from image volume to dense corresponding surface geometries provides isometric triangulated meshes (approximate edge length of 1.5 mm) with a segmentation accuracy close to pixel size (Audenaert et al., 2019b). The SSM behind these tasks is composed of dense corresponding meshes based on more than 600 lower limb segmentations and was previously validated with regards to population coverage, sexual dimorphism, model specificity, generalizability, and accuracy (Styner et al., 2003; Audenaert et al., 2019b). Further, and compared to manual processing, this model based automation was shown to reduce the data pre-processing effort with a factor 50, basically from hours to minutes (Audenaert et al., 2019b). SSMs of the hip articulation as well as isolated bony structures were used.

In order to define the corresponding cartilage geometry on the surface of the skeletal anatomy, the articulating cortical vertices were isolated and projected according to their surface normal. Cortical surfaces vertices were defined as articulating when covered by cartilaginous tissue on CT arthrography (Harris et al., 2012). Corresponding surfaces of overlying articular cartilage were thereby defined for both the acetabular and femoral components of the joint. The distance over which the cartilage was projected depended on the particular cartilage geometry prediction method that was implemented. Details on this are elaborated in section “Assignment of Cartilage Thickness to the Discrete Element Analysis Models.”

#### Construct of the Discrete Element Model

A custom MATLAB (R2016a, MathWorks, Natick, MA, United States) script was written to perform DEA. The DEA model was defined by two distinct layers, one representing the acetabular cartilage and a second layer representing the femoral cartilage. Each spring in the model was attached to a cortical vertex and its corresponding, projected, cartilage vertex, thereby representing nodal cartilage thickness for each layer of cartilage. As such this represents the major difference with FEA since by using spring elements only compression in the normal direction is considered and tangential and binormal deformations are neglected. Springs were activated in the region where the femoral head cartilage was in contact with the acetabular cartilage and were defined to resist only compressive forces. In DEA, springs representing cartilage are commonly assumed to exhibit linear stress behavior. However, experimental studies have demonstrated that articular cartilage exhibits non-linear mechanical behavior (Ateshian et al., 1997). Therefore, both linear and non-linear springs were evaluated. For the linear spring definition, the force, *F*, generated by compression of an individual spring *i*, was calculated as follows:

(1) $$F_{i}=k\varepsilon_{i}n_{i}S_{i}$$

with

(2) $$\varepsilon_{i}=\frac{\Delta d_{i}}{h_{i}}\frac{l_{i}}{h_{i}}$$

where *k* is the spring stiffness, ε*_*i*_* the strain, *n*_*i*_ the articular surface normal and *S*_*i*_ the triangular element area (Eq. 1). The strain, ε*_*i*_*, of each spring in the acetabular or femoral cartilage layer was defined by the compression distance, Δ*d*_*i*_, combined thickness of both cartilage layers, *h*_*i*_, and the thickness of the cartilage layer the spring belongs to, *l*_*i*_ (Eq. 2). The spring stiffness, *k*, was estimated from the cartilage Poisson’s ratio, *v*, and Young’s modulus, *E*, using data from the literature (*v* = 0.45 and *E* = 11.85 MPa; Eq. 2) (Genda et al., 2001; Yoshida et al., 2006; Abraham et al., 2013):

(3) $$k=\frac{E\left(1-v\right)}{\left(1-2v\right)\left(1+v\right)}$$

The force, *F*, generated from the non-linear spring definition was based on previous research (Volokh et al., 2007), and was calculated as follows:

(4) $$F_{i}=H_{AO}\left(1-\varepsilon_{i}+\frac{3\left(1+4\beta \right)}{2}\varepsilon_{i}^{2}\right)\varepsilon_{i}n_{i}S_{i}$$

where *H*_*AO*_ and β are material parameters determined from experimental stress-strain curves in human and bovine cartilage (*H_*AO*_* = 0.40 and β = 0.35) (Ateshian et al., 1997; Huang et al., 2005; Volokh et al., 2007).

A residual function was defined as the absolute value of the difference between the total sum of spring forces of both layers and the hip joint reaction force. A gradient descent optimization algorithm was used to define the optimal translation of the femoral head in the acetabular socket to minimize the residual function. Cartilage contact stresses were calculated from the spring force and the surface area of the triangular faces adjacent to each spring attachment.

#### Verification of the Discrete Element Model

Verification is defined as the process of determining that a computational model accurately represents the underlying mathematical model and its solution (Viceconti et al., 2005; Anderson et al., 2007; Henninger et al., 2010). For this purpose, a linear-elastic boundary value problem formulation defined by Bartel et al. (1985) was used. The analytical model consisted of two rigid hemispheres with radii of 20 and 24 mm. A single elastic layer of 4 mm was conformed to the rigid hemispheres and represented an average combined thickness of acetabular and femoral cartilage (Menschik, 1997; Kohnlein et al., 2009). Displacement was assumed to occur only in the radial direction. The obtained analytical solution was compared with the DEA solution as well as previously calculated FEA result (Anderson et al., 2008; Abraham et al., 2013). While all DEA models were performed in MATLAB, all FE analyses were run in NIKE3D (Harris et al., 2012). In all models, cartilage was defined with equivalent material properties (*ν* = 0.45 and *E* = 11.85 MPa). The one layer analytical and FEA model were defined with a sliding interface between the smaller rigid hemisphere and the 4 mm thick cartilage (represented by 4 mm springs in the one-layer DEA). For the two-layer FEA model this interface was located between both cartilage layers (represented by two layers of 2 mm springs). Finally, a compressive force of 2000 N was applied, which was consistent with forces measured *in vivo* using telemeterized hip prostheses (Bergmann et al., 2016). Contact stress was calculated as a function of theta, the angle from vertical.

#### Validation of the Discrete Element Analysis Models

**FIGURE 1:**
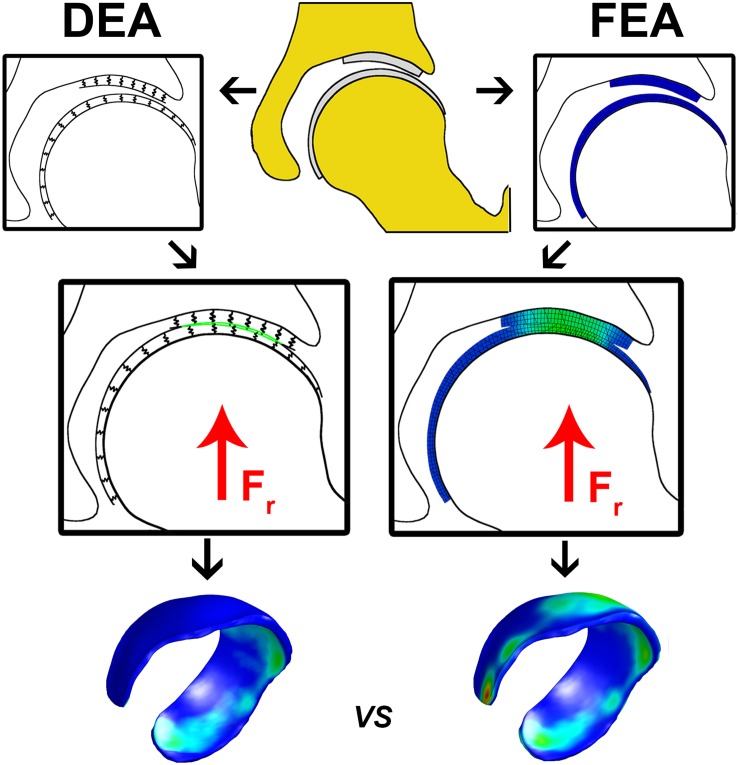
Flowchart illustrating the calculation of cartilage contact stresses using the DEA (left) and FEA (right) technique. The upper center figure represents hip anatomy segmented from CT arthrography images. In the DEA model the articular cartilage vertices were isolated and the distance between the rigid bone and the articulating cartilage layer was modeled by an array of springs representing the femoral and acetabular layers. In the FEA model, femoral and acetabular cartilage were modeled with three hexahedral elements through the thickness, which was previously shown to yield converged contact stress predictions (Anderson et al., 2008). A vertical compression force was applied to the FEA and DEA models and displacements in three directions were allowed for the femur until a steady state was reached. The DEA stresses at the acetabular cartilage were compared to those from the FEA model on a node-by-node basis. The DEA models were validated by comparing peak and average contact stresses as well as contact area with those predicted from the FEA models.

Validation is defined as the process of ensuring that the computational model accurately represents the physics of the real-world system. Herein, DEA models were validated using previously-published FEA models from ten asymptomatic, morphologically-normal volunteers (Figure 1; Harris et al., 2012). This group had an average ± standard deviation lateral center-edge angle of 33.5 ± 5.48 and an acetabular index of 4.6 ± 3.78°. Age, weight, and body mass index (BMI) were 26 ± 4 years, 70.0 ± 13.9 kg, and 23 ± 4 kg/m^2^, respectively. Geometry for the FEA models was derived from segmented CT arthrography images (120 kVp, 100–400 mAs, 512 × 512 matrix, 1.0 pitch, 300–400 mm FOV, 1.0-mm slice thickness).

The FEA models were analyzed under simulated walking at heel strike (WHS), ascending staircase at heel strike (AHS) and descending staircase at heel strike (DHS) using data from the literature (Bergmann et al., 2001). For each loading scenario, the pelvis was fixed in space. Load was applied to the femur in the direction and magnitude measured by Bergmann et al. (2001). The femur was free to translate to find a solution that minimized energy (Harris et al., 2012). Cortical bone and cartilage surfaces were discretized using triangular shell and hexahedral elements, respectively. Cartilage was modeled as a homogeneous, isotropic, nearly incompressible, neo-Hookean hyperelastic material with shear modulus *G* = 13.6 MPa and bulk modulus *K* = 1,359 MPa (Poisson’s ratio *ν* = 0.495) (Anderson et al., 2008). Cortical bone was modeled as a homogeneous, isotropic material with elastic modulus *E* = 17 GPa and Poisson’s ratio *ν* = 0.29. Mesh densities were determined from convergence studies (Anderson et al., 2008). All FE models were analyzed using NIKE3D (Lawrence Livermore Natl. Lab.; Livermore, CA, United States). Nodal stresses on the articular side of the acetabular cartilage were extracted from the FE models and were used as the reference standard for comparison of results from the corresponding DEA models.

A separate DEA model with two layers of springs representing the femoral and acetabular cartilage, respectively, was created for each of the ten subjects for which an FE model was also available. Each spring of the DEA model had a cartilage thickness equal to that of the FE model at that location. The DEA models assumed equivalent material properties for cartilage as the FE models and were loaded in the same manner. However, the DEA models assumed a rigid subchondral bone-cartilage interface (i.e., rigid bones). Analyses were conducted using both linear and non-linear springs. Root mean squared (RMS) and absolute differences in contact stresses, predicted by the DEA models compared to FE results, were evaluated on a node-by-node basis. In addition, peak contact stress, average contact stress and contact area predicted by DEA were compared to FE results using Bland-Altman plots. Here, the cartilage contact area was defined as the sum of the surface area of the mesh triangle connected by the acetabular cartilage nodes that were in contact with the femoral cartilage. The average contact stress was calculated from the acetabular cartilage nodes that were in contact with the femoral cartilage; areas where cartilage was not in contact were not considered in the calculation of average stress.

### Assignment of Cartilage Thickness to the Discrete Element Analysis Models

**FIGURE 2:**
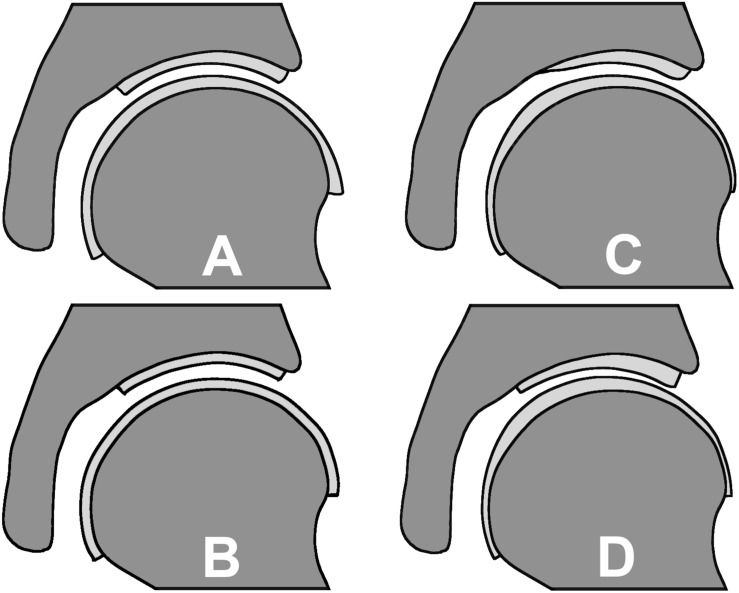
Cartilage geometry prediction methods: **(A)** spherical method resulting in a varying cartilage thickness, **(B)** constant cartilage thickness method, **(C)** nodal thickness method which predicts a varying cartilage thickness based on an average cartilage thickness distribution map and **(D)** the manually segmented subject-specific cartilage as the reference.

As mentioned above, computational modeling of hip contact mechanics could become more efficient if the cartilage anatomy could be estimated, rather than segmented. To this end, two commonly-used (sphere fitting, calculation of a constant cartilage thickness) and one novel method (assignment of population-averaged cartilage thickness) were used to assign cartilage thickness to the DEA models (see Figure 2). Each method used the geometry of the outer cortex of the bone as the basis for estimating cartilage thickness. Further details on each method are given in the following subsections.

#### Spherical Fitting Technique

For this methodology, anatomy representing the outer cortex of the acetabulum and femoral head was used to estimate a respective average acetabular and femoral radius *R*_*a*_ and *R*_*f*_ by means of least squares spherical fitting of the articulating surfaces. The joint radius, *R*, separating the femoral cartilage from the acetabular cartilage was then determined as follows

(5) $$R=\frac{R_{a}+R_{f}}{2}$$

This joint radius was then projected back to the underlying subject-specific bony anatomy (Anderson A. E. et al., 2010). Using this approach made the articulating interfaces congruent, but still provided a location-specific cartilage thickness for each DEA spring.

#### Constant Cartilage Thickness

The constant thickness definition added a cartilage layer to the subject-specific cortical surface of the acetabulum and femoral head. Here, the cartilage thickness, *d* remained constant and was calculated as:

(6) $$d=\frac{R_{a}-R_{f}}{2}$$

Using this approach, the femoral and acetabular cartilage layers were assigned the same thickness. Thus, the geometry of the articulating surface was simply an extrusion of the underlying cortex along the direction of the surface normals, yielding a contact interface that was not congruent.

#### Population-Averaged Cartilage Thickness Map

Similar to the constant thickness method, this approach modeled the cartilage thickness by extruding the outer cortex of the femoral and acetabular bone surface nodes along the direction of the surface normal. However, instead of a constant thickness for all nodes, each node exhibited a location-specific thickness determined based on manually-segmented cartilage layers of the hip joint for 10 subjects (Harris et al., 2012). Before calculating the population average of nodal cartilage thickness, the local cartilage thickness values were scaled corresponding to the radius of the matching femoral head, which was determined from a best-fit sphere of the articulating surface. The resultant population-averaged cartilage thickness maps therefore exhibited distance properties that allowed for nodal-based estimation of cartilage geometry for any new subject of interest according to local hip morphometrics.

#### Validation of Cartilage Geometry Prediction

To evaluate the accuracy of cartilage geometry predictions, the three prediction methods were compared to the manually segmented subject specific cartilage by evaluating the distance from each node of the predicted cartilage to the subject-specific cartilage layer. The RMS error (RMSE) and maximal error were calculated to evaluate the geometric mismatches. One-way Anova was used to identify statistically significant differences. For the validation of the population-averaged cartilage geometry, a k-fold leave-one-out methodology was used to avoid including prior anatomical knowledge to the prediction of the cartilage geometry.

### Impact of Cartilage Geometry Definitions on DEA Contact Mechanics

Cartilage geometry, obtained by manual segmentation of volumetric images in concert with FEA, remains the gold standard to simulate hip contact mechanics. However, this methodology is not realistic in the setting of large population studies. Therefore, use of an approximation method to define cartilage geometry, when combined with DEA, could substantially increase the efficiency at which cartilage stresses are predicted. Still, whether improvements on cartilage geometry predictions as opposed to commonly used generic definitions (e.g., spherical fit or constant thickness) significantly impacts on contact stress predictions remains to be demonstrated. The purpose of this section is therefore to evaluate and benchmark the combined use of cartilage geometry prediction techniques and the simplified DEA approach, against the gold standard of FEA using manual cartilage segmentation.

Femoral and pelvic anatomy were derived from 10 subjects from which manual segmentations were available (Harris et al., 2012). These manual segmented surface representations were then fitted within the statistical shape model, to allow the geometric morphometric pipeline to be followed. The previously described shape model was fitted with increasing degrees of freedom (by gradually increasing the principal components) onto the manual segmented cortical surfaces by iteratively solving an overdetermined set of linear regression equations in a least squares sense, while adapting for pose (rotation, translation, scale) changes using singular value decomposition. We refer to Audenaert et al. (2019b) for full details on the fitting procedure.

Following, each cartilage geometry prediction method was implemented in distinct DEA models to represent the hip joints of the 10 subjects for which subject-specific cartilage reconstructions were available (Harris et al., 2012). Next, the same AHS, DHS and WHS loading scenarios and boundary conditions were applied to both the subject-specific FEA models and the aforementioned DEA models (see section “Construct of the Discrete Element Model”) (Harris et al., 2012).

The difference between DEA contact stress predictions of the spherical fit, constant thickness and population averaged cartilage geometry and the FEA contact stress results with manually segmented cartilage geometry was calculated. For this purpose, nearest neighbor correspondence was established between the predicted cartilage layers and the subject-specific cartilage layer, which allowed node-to-node comparisons of the contact stresses. Peak contact stress, average contact stress and contact area were also evaluated using Bland-Altman plots. RMSE and maximum error were reported.

## Results

### Discrete Element Analysis

#### Verification

**FIGURE 3:**
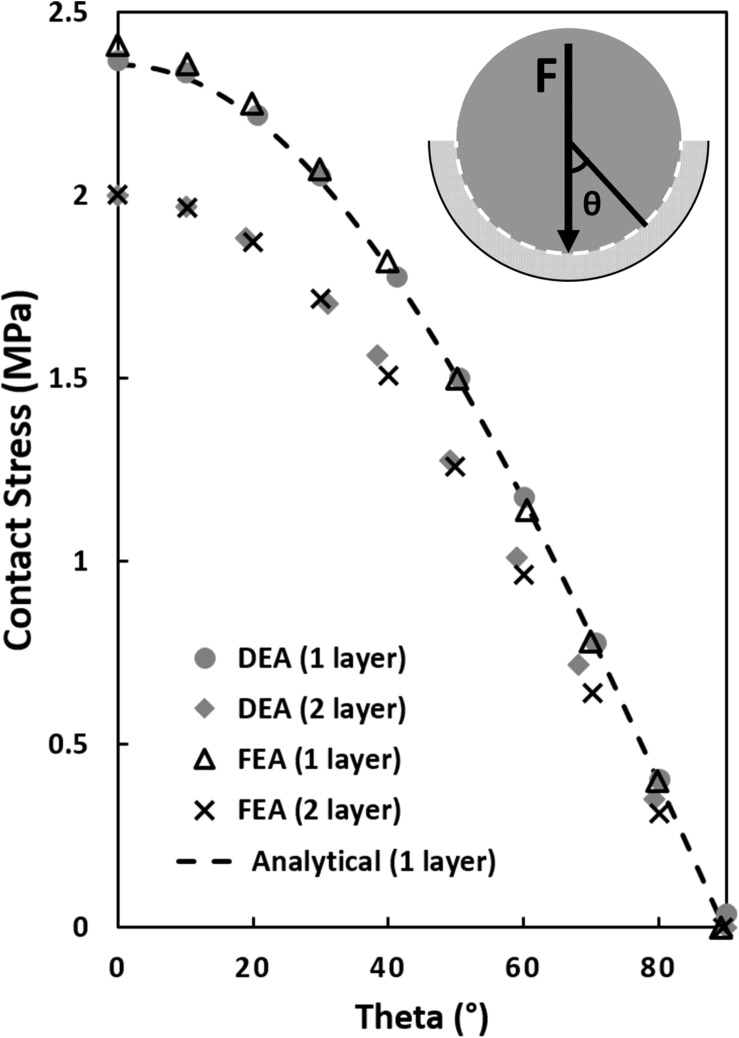
Contact stress calculations in a simplified ball and socket joint with a one and two elastic layer configuration. The analytical one-layer elastic model featured a 20 mm rigid ball/hemisphere compressing a 4 mm thick elastic layer backed by a 24 mm rigid shell (see top right corner, sliding interface is modeled between the 20 mm rigid sphere and the elastic layer). The two-layer FEA and DEA models consisted of two elastic layers with the sliding interface between the layers. Contact stress was plotted as a function of theta, θ, the angle from vertical. The equivalent one-layer FEA and DEA model solutions were similar to the analytical model but 18% higher than the two-layer models at the location of maximum contact stress (θ = 0°).

The two-layer DEA model predicted contact stresses that were very similar to the two-layer FEA model (RMSE = 0.007 MPa). The one-layer FEA and one-layer DEA solutions were approximately equal to the analytical one-layer calculation (RMSE = 0.019 and 0.016 MPa, respectively). For both FEA and DEA, contact stresses estimated by the one-layer models were higher than the two-layer models. Differences were largest at the location of peak contact stress (θ = 0°), where the one layer models predicted a peak contact stress 18% higher than the two layer models (see Figure 3).

#### Validation

**FIGURE 4:**
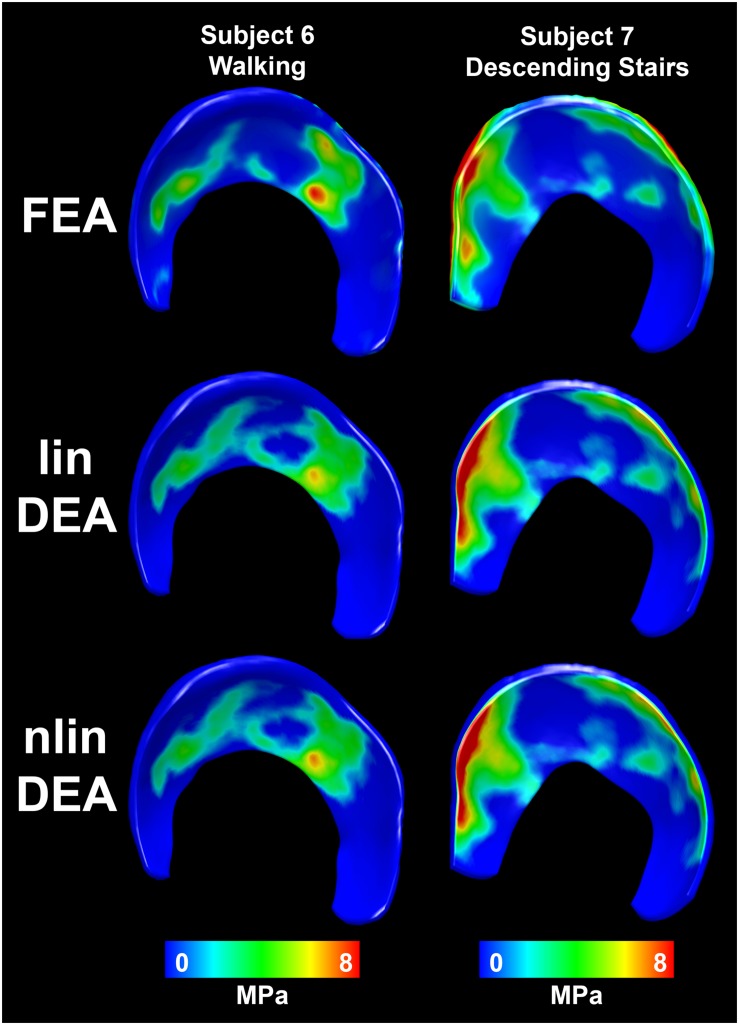
Distribution of contact stresses on the acetabular cartilage estimated with the reference FEA (top row), linear DEA (middle row) and non-linear DEA (bottom row). The overall contact patterns as well as the magnitude of the contact stresses corresponded well between both DEA techniques and FEA. Identical cartilage geometry, obtained by manual segmentation for each subject, was used in all three numerical methods.

**FIGURE 5:**
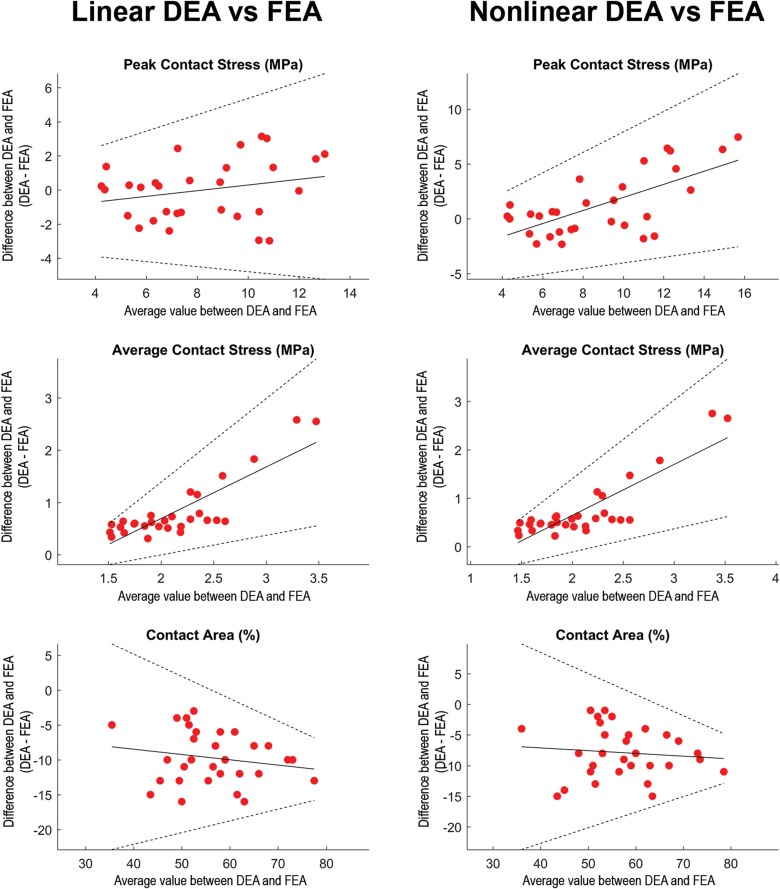
Regression-based Bland-Altman plots comparing linear DEA (column 1) and non-linear DEA (column 2) with FEA. The difference (*DEA*_*i*_–*FEA*_*i*_) between both methods was plotted as a function of the average $\left(\frac{DEA_{i}-FEA_{i}}{2}\right)$ for each of the loading scenarios (represented by dots). Solid lines represent the regression line and its 95% confidence limits (Bland and Altman, 1999). In loading situations with high articular peak stresses calculated with FEA, the non-linear DEA tended to overestimate peak stress. Overall, the average contact stresses (middle row) were higher in the DEA models. In loading scenarios with high average contact stresses, both linear as well as non-linear DEA increasingly overestimated average contact stresses. The contact area (lower row) from the linear and non-linear DEA models consistently under-predicted contact area estimated by FEA. Identical cartilage geometry, obtained by manual segmentation for each subject, was used in all three numerical methods.

Unless otherwise noted, all results were presented as average ± standard deviation. The overall patterns of contact for the DEA models corresponded well with the FE models for all loading scenarios (Figure 4). Contact areas for DEA were −9.7 ± 3.8% smaller for the linear definition and −7.9 ± 4.1% for the non-linear definition. Peak contact stresses corresponded well, with an average difference of −0.01 ± 1.77 MPa for the linear definition. The non-linear DEA model overestimated the articular peak stress by 1.24 ± 2.87 MPa. The overestimation of the non-linear model increased in high loading scenarios as these involve areas of higher cartilage strain (Figure 5). The reduced contact area in the DEA results was accompanied by an increased average contact stress which was 0.83 ± 0.57 MPa higher for the linear and 0.74 ± 0.63 MPa higher for the non-linear DEA model compared to the FEA solution (overall FEA average contact stress = 1.72 MPa). The node-based stress difference was on average 0.18 ± 0.16 MPa higher in the linear DEA and 0.18 ± 0.18 MPa higher in the non-linear DEA compared to FEA.

**FIGURE 6:**
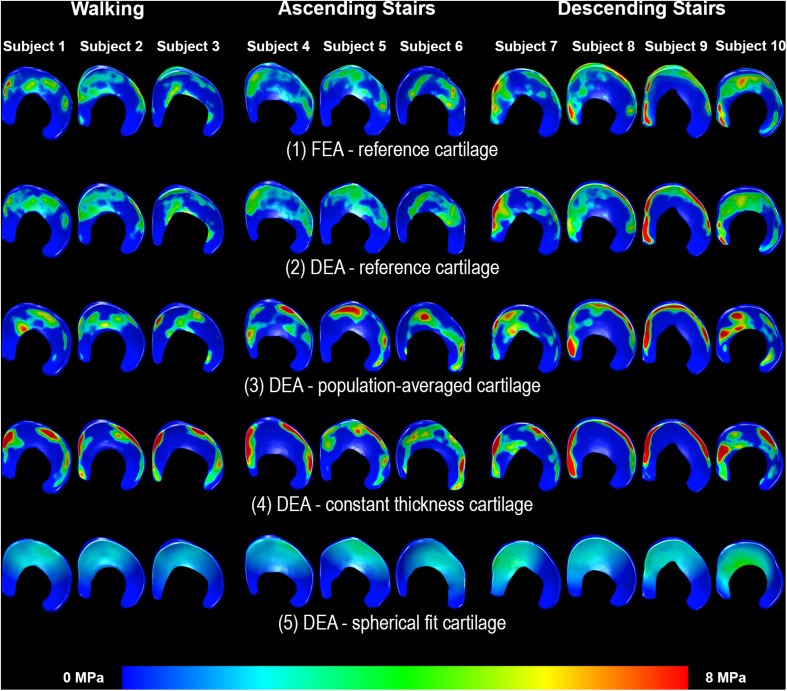
Distribution of contact stresses on the acetabular cartilage during walking (subjects 1–3), ascending stairs (subjects 4–6) and descending stairs (subjects 7–10). Left side shows the anterior horn of the acetabular cartilage. Each column represents one subject. Row 1 displays the results from the FEA analysis with the reference subject-specific cartilage geometry. The linear DEA using reference cartilage geometry (row 2) layers reveals results similar to FEA. Results from the DEA models using the three methods to predict cartilage thickness are shown in rows 3–5. As can be seen, DEA predictions from the population-averaged cartilage thickness method were most like the reference DEA and FEA models. Notably, the constant thickness model resulted in highly concentrated stresses, whereas the spherical model under-estimated stresses and predicted a more diffuse and uniform contact pattern.

The contact stress distributions corresponded very well between the FEA and DEA solutions, despite differences in contact area and stresses (Figure 6). This was illustrated by an average node-to-node stress difference limited to 0.18 MPa (±0.17 MPa). Furthermore, the difference in femur translation required to balance the reaction force was on average 0.3 mm (±0.17 mm) between both techniques. This residual difference in equilibrium position might also be attributed to the lack of Poisson’s effect in the DEA spring model.

**FIGURE 7:**
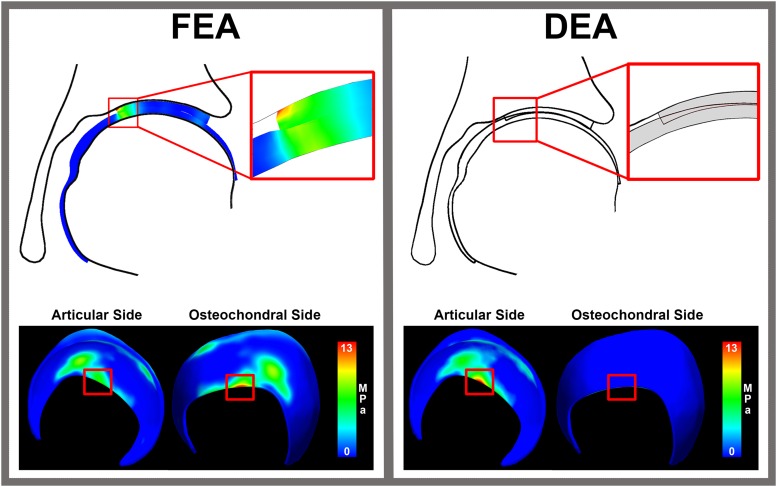
Cartilage stresses in an edge loading situation. The distribution of stress throughout cartilage layer in FEA reveals peak stresses around 13 MPa at the osteochondral cartilage side. In an edge loading situation, the highest stresses calculated with FEA were located at the osteochondral surface instead of the articular surface. This may in part explain the discrepancy between DEA and FEA results, since only the contact stresses at the articulating surface of the FEA models were used to compare to the DEA results of the articular side.

**FIGURE 8:**
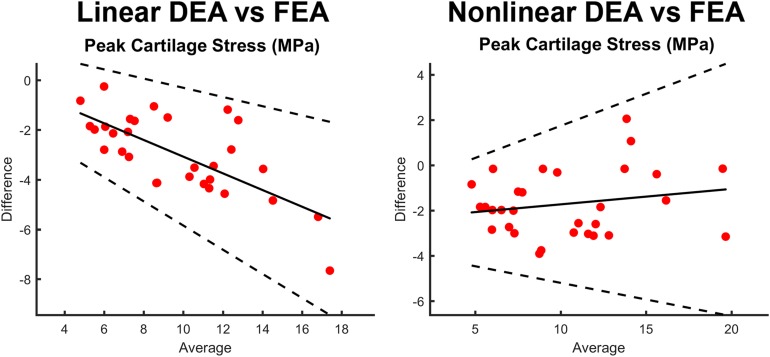
Bland-Altman plots comparing peak stress through the entire thickness of cartilage in the FEA model as compared to the contact stress reported by the linear DEA (left) and non-linear DEA (right) models. The non-linear and linear DEA models yielded similar predictions in cases with low peak stresses. However, in loading positions with high peak stresses, the non-linear DEA definition yielded more accurate predictions.

In edge loading situations, the highest stresses calculated with FEA were located at the osteochondral surface instead of the articular surface (Figure 7). This may in part explain the discrepancy between DEA and FEA results, since only the contact stresses at the articulating surface of the FEA models were used to assess the accuracy of DEA. To help us better-understand this effect, we compared peak contact stress from the DEA model to the peak cartilage stress through the entire thickness of cartilage (including the osteochondral boundary) from the FEA model, and found better agreement (Figure 8).

### Accuracy of Cartilage Geometry Predictions

**FIGURE 9:**
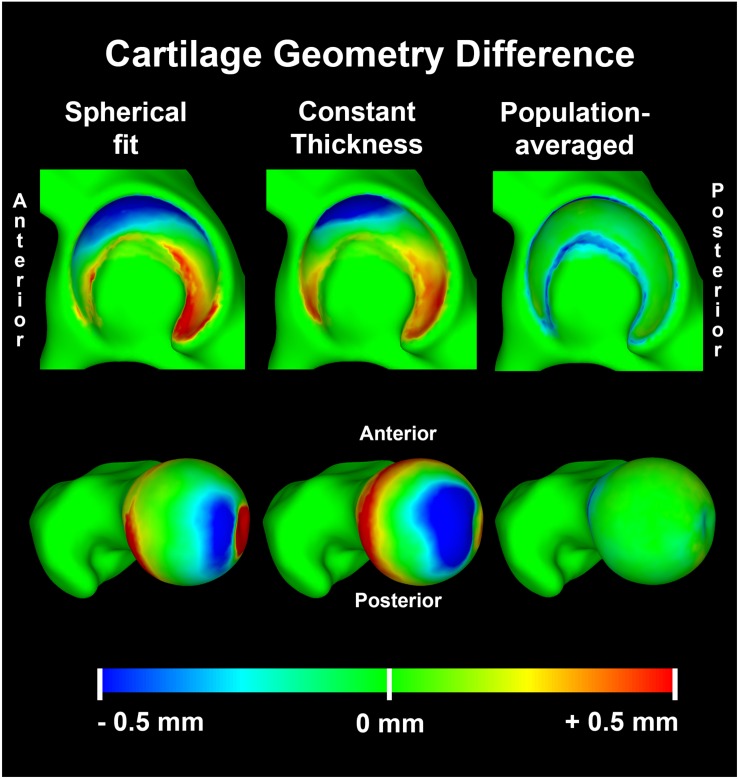
Average distance difference in predicted cartilage geometry compared to the reference cartilage geometry visualized on a template acetabulum and proximal femur. The population averaged method provided the most accurate predictions of cartilage thickness, with errors confined to the periphery of both the acetabular and femoral cartilage.

The RMSE of the spherical and constant thickness cartilage geometry compared to the reference cartilage geometry was 0.46 ± 0.11 and 0.48 ± 0.11 mm, respectively. When using the population-averaged nodal thickness method errors decreased to 0.31 ± 0.08 mm. The observed differences were statistically significant (*p* < 0.001). The maximum error was also lowest when using the population-averaged method (1.59 ± 0.27 mm). An overview of the findings is presented in Table 1. Both the spherical and constant thickness prediction method grossly underestimated cartilage thickness at the superolateral acetabulum and superomedial femur and overestimated cartilage thickness at the anterior and posterior horn of the acetabulum. The population-averaged resulted in the best overall approximation of the subject-specific cartilage with mostly underestimations confined to the periphery of the acetabular and femoral cartilage (Figure 9).

**TABLE 1:** Summary of cartilage thickness and DEA model errors using three methods to define cartilage anatomy.

	Spherical	Constant thickness	Population-averaged
**Cartilage thickness**			
RMSE (mm)	0.46 ± 0.11	0.48 ± 0.11	0.31 ± 0.08*
Maximal error (mm)	2.87 ± 1.49	2.10 ± 0.50	1.59 ± 0.27*
**Stress analysis (DEA-FEA)**			
Peak Stress (MPa)	−5.48 ± 2.33	8.77 ± 5.49	1.68 ± 2.63
Average contact stress (MPa)	−0.42 ± 0.20	2.87 ± 1.23	1.46 ± 0.63
Contact area (%)	14.6 ± 11.0	−25.3 ± 11.3	−20.6 ± 7.4

Errors represent the difference in distance between the predicted cartilage geometry versus the reference cartilage geometry based on reconstructions of CT arthrography images. DEA model errors represent the aggregate for all loading scenarios examined. * statistically significant difference at the 0.001 level between population-averaged method compared to the spherical and constant thickness method, respectively.

### Impact of Cartilage Geometry Definitions on DEA Results

Although the accuracy to predict cartilage geometry was improved using the population-averaged cartilage geometry, the clinical relevance in terms of improved estimation of contact mechanics remains the most important question. For the linear DEA models, the constant thickness cartilage geometry overestimated peak stresses by 8.77 ± 5.49 MPa compared to the subject-specific FEA models. In contrast, the spherical definition resulted in an average underestimation of −5.48 ± 2.33 MPa. The population averaged cartilage geometry was closest to the FEA model results with an average difference in peak stress of 1.68 ± 2.63 MPa. Overall difference in average contact stress was low between the different methods, with the highest difference found with constant thickness method (2.87 ± 1.23 MPa). A detailed overview of the findings for the different methods is presented in Table 1.

## Discussion

With larger datasets becoming available, in combination with increased computational resources, statistical and probabilistic modeling presents an exciting means for non-invasive testing and the evaluation of physiology and biomechanical variability across populations. In the present study we present a novel cartilage anatomy prediction method that builds on geometric morphometrics that can be integrated with discrete element models. The described method does not require the cartilage of each subject to be manually segmented, and thus, shows promise for mechanical analysis of large sample sizes to clarify the pathogenesis of hip OA in patients with structural hip disease.

In this study, we demonstrated that the novel population-averaged prediction method to estimate cartilage geometry for DEA models yielded cartilage contact mechanics that were in better agreement with subject-specific FEA models when compared to the spherical fit or constant thickness methods. Previous studies demonstrated that parameterized models working with idealized geometries consistently underestimate contact stresses (Anderson A. E. et al., 2010; Gu et al., 2011), however the presented methodology seems to provide a valid alternative, as it provides more accurate estimations of cartilage anatomy without requiring segmentation.

In all DEA models evaluated in the present work, contact stresses biased toward higher magnitudes than FEA, with 45% higher average contact stresses and 9% less contact area. These results agreed with previous studies comparing the performance of DEA to FEA. In particular, Abraham et al. implemented a one-layer DEA method to mimic the anatomy of a subject-specific FE model of the hip (Abraham et al., 2013). Similar to our results herein, Abraham et al. found that the average contact stresses were 43% higher and contact area was 10% lower in the DEA model as compared to FEA. In general little difference in outcome between the one- and two-layer approach was observed. The main advantage of the two-layer approach is that joint stresses could be evaluated separately on the respective joint surfaces as opposed to the one layer definition. A DEA study of the ankle following articular fractures compared a two-layer DEA model to FEA, and found that average contact stresses were 12% higher with a contact area that was 3.2% lower compared to FEA, indicating a similar trend to the results by Kern and Anderson (2015).

In general the linear DEA models performed similar to the non-linear models. This is in agreement with previous work by Volokh et al. (2007). However, in loading situations with high articular peak stresses calculated with FEA, the non-linear DEA peak stresses were found to be even higher. In these edge loading situations, the highest stresses calculated with FEA were located at the osteochondral surface instead of the articular surface. This may in part explain the discrepancy between DEA and FEA results, since only the contact stresses at the articulating surface of the FEA models were used to assess the accuracy of DEA.

The importance of the cartilage geometry in the analysis of cartilage contact mechanics cannot be underestimated. Cadaveric tests using pressure-sensitive film demonstrate peak pressures ranging from 7 MPa at 50% BW to 10 MPa at 350% BW, with irregular contact stress distributions over the articulating surface (Afoke et al., 1987; von Eisenhart-Rothe et al., 1997; Anderson et al., 2008). Although a spherical cartilage assumption has often been applied for evaluation of contact mechanics, our results confirmed that the idealized cartilage geometry inflicts an overestimation of contact area and fails to predict realistic contact stress distributions. Consequently, peak stresses from the spherical model were much lower than those from the subject-specific DEA and FEA models. The inclusion of subject-specific geometry of the bony contours with a constant thickness layer of cartilage resulted in an irregular contact pattern that better approximated the FEA subject-specific patterns than the spherical approach. However, peak stresses from the constant thickness model were double those of the subject-specific DEA and FEA models. These findings were in agreement with a previous study that used a constant cartilage thickness FEA model (Anderson A. E. et al., 2010). Hip cartilage shows regional variations in thickness that were not accurately represented when using the sphere fitting and constant cartilage thickness DEA models (Adam et al., 1998; Anderson et al., 2008). Conversely, the population-averaged cartilage method incorporated a distribution of cartilage thicknesses and modeled the underlying subject-specific geometry at the osteochondral interface. As a result, stress distribution patterns better-matched the reference FEA subject-specific models and peak stress overestimation was limited to 20%. The contact area was on average 21% lower with average contact stresses that were 85% higher than the FEA solution, thereby substantially outperforming the constant thickness method (25% lower contact area and 166% higher average contact stresses).

There were a number of limitations to our study. First, the DEA model did not include the labrum, which has been shown to carry load and is thus a relevant structure when quantifying hip contact mechanics. Nevertheless, in the context of detecting high loading areas, the absent labrum does not pose problems since it has been shown that the location of peak stresses is similar in FEA models with and without labrum (Henak et al., 2014). Furthermore, the labrum was not included in either the DEA or FEA models, and thus, the results from DEA and FEA in this study are directly comparable. Second, the FEA model assumed deformable bones, but all DEA models analyzed herein assumed rigid bones. This choice was made for ease of use and simplicity of the models. Theoretically it is perfectly possible to include deformation of the cortical bone by assigning composite material properties to the spring element (Anderson D. D. et al., 2010). We chose to allow bones to be deformable in the FEA model, as it represents the current state-of-the-art, making it serve as an ideal reference standard in which to quantify the accuracy of FEA. Future studies could assume rigid bones for FEA or devise new techniques to account for bone deformation in DEA. A specific limitation of the population-averaged cartilage map is the fact that it is – for now- based on the anatomy of morphologically-normal volunteers and is therefore best to be used in this subset of the population. K-fold leave-one-out validation of the cartilage prediction method performed well in this subset. In the future adding cartilage geometries from dysplasia and other hip morphologies will be required in order to study patients with structural hip disease. Finally, the correspondence model provided the means to compare stress results from the DEA models that used the three methods to predict cartilage thickness. Naturally, there will be some errors when comparing results using this approach, as the location of each DEA spring was different between the three models.

## Conclusion

We found that use of a population-averaged cartilage method in combination with DEA provided a suitable alternative compared to subject-specific FEA models. When applying DEA models, users should keep in mind that it consistently underestimates contact area and overestimates peak and average contact stress. The linear DEA robustly estimated articular contact pressures, whereas the non-linear definition was more sensitive to high peak stresses throughout the full cartilage layer in edge loading situations. While the goal of the modeling study will ultimately dictate the accuracy needed for estimating hip cartilage contact stresses, our results for DEA with the population-averaged method are encouraging, as this technique could be used to analyze cartilage contact stresses in a large sample size without the need to segment cartilage, and with much greater computational efficacy as compared to FEA.

## Data Availability Statement

The datasets generated for this study are available on request to the corresponding author.

## Author Contributions

All authors listed have made a substantial, direct and intellectual contribution to the work, and approved it for publication.

## Conflict of Interest

The authors declare that the research was conducted in the absence of any commercial or financial relationships that could be construed as a potential conflict of interest.

## References

[B1] Abraham C. L. Maas S. A. Weiss J. A. Ellis B. J. Peters C. L. Anderson A. E. (2013). A new discrete element analysis method for predicting hip joint contact stresses. *J. Biomech.* 46 1121–1127. 10.1016/j.jbiomech.2013.01.012 23453394 PMC3623562

[B2] Adam C. Eckstein F. Milz S. Putz R. (1998). The distribution of cartilage thickness within the joints of the lower limb of elderly individuals. *J. Anat.* 193 203–214. 10.1046/j.1469-7580.1998.19320203.x 9827636 PMC1467840

[B3] Afoke N. Y. Byers P. D. Hutton W. C. (1987). Contact pressures in the human hip joint. *J. Bone. Joint. Surg. Br.* 69 536–541. 10.1302/0301-620x.69b4.3611154 3611154

[B4] Agricola R. Heijboer M. P. Bierma-Zeinstra S. M. A. Verhaar J. A. N. Weinans H. Waarsing J. H. (2013a). Cam impingement causes osteoarthritis of the hip: a nationwide prospective cohort study (CHECK). *Ann. Rheum. Dis.* 72 918–923. 10.1136/annrheumdis-2012-201643 22730371

[B5] Agricola R. Heijboer M. P. Roze R. H. Reijman M. Bierma-Zeinstra S. M. A. Verhaar J. A. N. (2013b). Pincer deformity does not lead to osteoarthritis of the hip whereas acetabular dysplasia does: acetabular coverage and development of osteoarthritis in a nationwide prospective cohort study (CHECK). *Osteoarthr. Cartil.* 21 1514–1521. 10.1016/j.joca.2013.07.004 23850552

[B6] Almeida D. F. Ruben R. B. Folgado J. Fernandes P. R. Audenaert E. Verhegghe B. (2016). Fully automatic segmentation of femurs with medullary canal definition in high and in low resolution CT scans. *Med. Eng. Phys.* 38 1474–1480. 10.1016/j.medengphy.2016.09.019 27751655

[B7] Anderson A. E. Ellis B. J. Maas S. A. Peters C. L. Weiss J. A. (2008). Validation of finite element predictions of cartilage contact pressure in the human hip joint. *J. Biomech. Eng.* 130:051008. 10.1115/1.2953472 19045515 PMC2840996

[B8] Anderson A. E. Ellis B. J. Maas S. A. Weiss J. A. (2010). Effects of idealized joint geometry on finite element predictions of cartilage contact stresses in the hip. *J. Biomech.* 43 1351–1357. 10.1016/j.jbiomech.2010.01.010 20176359 PMC2857573

[B9] Anderson D. D. Iyer K. S. Segal N. A. Lynch J. A. Brown T. D. (2010). Implementation of discrete element analysis for subject-specific, population-wide investigations of habitual contact stress exposure. *J. Appl. Biomech.* 26 215–223. 10.1123/jab.26.2.215 20498493 PMC2905528

[B10] Anderson A. E. Ellis B. J. Weiss J. A. (2007). Verification, validation and sensitivity studies in computational biomechanics. *Comput. Methods Biomech. Biomed. Eng.* 10 171–184. 10.1080/10255840601160484 17558646 PMC3361760

[B11] Anderson L. A. Anderson M. B. Kapron A. Aoki S. K. Erickson J. A. Chrastil J. (2016). The 2015 frank stinchfield award: radiographic abnormalities common in senior athletes with well-functioning hips but not associated with osteoarthritis. *Clin. Orthop. Relat. Res.* 474 342–352. 10.1007/s11999-015-4379-6 26054483 PMC4709310

[B12] Ateshian G. A. Warden W. H. Kim J. J. Grelsamer R. P. Mow V. C. (1997). Finite deformation biphasic material properties of bovine articular cartilage from confined compression experiments. *J. Biomech.* 30 1157–1164. 10.1016/s0021-9290(97)85606-0 9456384

[B13] Audenaert E. A. Pattyn C. Steenackers G. De Roeck J. Vandermeulen D. Claes P. (2019a). Statistical shape modeling of skeletal anatomy for sex discrimination: their training size, sexual dimorphism, and asymmetry. *Front. Bioeng. Biotechnol.* 7:302. 10.3389/fbioe.2019.00302 31737620 PMC6837998

[B14] Audenaert E. A. Van Houcke J. Almeida D. F. Paelinck L. Peiffer M. Steenackers G. (2019b). Cascaded statistical shape model based segmentation of the full lower limb in CT. *Comput. Methods Biomech. Biomed. Eng.* 22 644–657. 10.1080/10255842.2019.1577828 30822149

[B15] Audenaert E. A. Van den Eynde J. de Almeida D. F. Steenackers G. Vandermeulen D. Claes P. (2020). Separating positional noise from neutral alignment in multicomponent statistical shape models. *Bone Rep.* 12:100243. 10.1016/j.bonr.2020.100243 32181268 PMC7063239

[B16] Bartel D. L. Burstein A. H. Toda M. D. Edwards D. L. (1985). The effect of conformity and plastic thickness on contact stresses in metal-backed plastic implants. *J. Biomech. Eng.* 107 193–199. 10.1115/1.3138543 4046559

[B17] Bergmann G. Bender A. Dymke J. Duda G. Damm P. (2016). Standardized Loads Acting in Hip Implants. *PLoS One* 11:e0155612. 10.1371/journal.pone.0155612 27195789 PMC4873223

[B18] Bergmann G. Deuretzbacher G. Heller M. Graichen F. Rohlmann A. Strauss J. (2001). Hip contact forces and gait patterns from routine activities. *J. Biomech.* 34 859–871. 10.1016/s0021-9290(01)00040-9 11410170

[B19] Bland J. M. Altman D. G. (1999). Measuring agreement in method comparison studies. *Stat. Methods Med. Res.* 8 135–160. 10.1191/096228099673819272 10501650

[B20] Ganz R. Leunig M. Leunig-Ganz K. Harris W. H. (2008). The etiology of osteoarthritis of the hip: an integrated mechanical concept. *Clin. Orthop Relat. Res.* 466 264–272. 10.1007/s11999-007-0060-z 18196405 PMC2505145

[B21] Genda E. Iwasaki N. Li G. A. MacWilliams B. A. Barrance P. J. Chao E. Y. S. (2001). Normal hip joint contact pressure distribution in single-leg standing - effect of gender and anatomic parameters. *J. Biomech.* 34 895–905. 10.1016/s0021-9290(01)00041-0 11410173

[B22] Gu D. Y. Hu F. Wei J. H. Dai K. R. Chen Y. Z. (2011). “Contributions of non-spherical hip joint cartilage surface to hip joint contact stress,” in *Proceedings of the Annual International Conference of the IEEE Engineering in Medicine and Biology Society. IEEE Engineering in Medicine and Biology Society*, (Piscataway, NJ: IEEE), 8166–8169. 10.1109/IEMBS.2011.6092014 22256237

[B23] Harris M. D. Anderson A. E. Henak C. R. Ellis B. J. Peters C. L. Weiss J. A. (2012). Finite element prediction of cartilage contact stresses in normal human hips. *J. Orthop. Res.* 30 1133–1139. 10.1002/jor.22040 22213112 PMC3348968

[B24] Henak C. R. Anderson A. E. Weiss J. A. (2013). Subject-specific analysis of joint contact mechanics: application to the study of osteoarthritis and surgical planning. *J. Biomech. Eng.* 135:021003. 10.1115/1.4023386 PMC3705883 23445048

[B25] Henak C. R. Ateshian G. A. Weiss J. A. (2014). Finite element prediction of transchondral stress and strain in the human hip. *J. Biomech. Eng.* 136:021021. 10.1115/1.4026101 PMC5101027 24292495

[B26] Henninger H. B. Reese S. P. Anderson A. E. Weiss J. A. (2010). Validation of computational models in biomechanics. *Proc. Inst. Mech. Eng. H* 224 801–812. 20839648 10.1243/09544119JEIM649 PMC2941217

[B27] Hosnijeh F. S. Zuiderwijk M. E. Versteeg M. Smeele H. T. W. Hofman A. Uitterlinden A. G. (2017). Cam deformity and acetabular dysplasia as risk factors for hip osteoarthritis. *Arthr. Rheumatol.* 69 86–93. 10.1002/art.39929 27696746

[B28] Huang C.-Y. Stankiewicz A. Ateshian G. A. Mow V. C. (2005). Anisotropy, inhomogeneity, and tension–compression nonlinearity of human glenohumeral cartilage in finite deformation. *J. Biomech.* 38 799–809. 10.1016/j.jbiomech.2004.05.006 15713301 PMC3786419

[B29] Kern A. M. Anderson D. D. (2015). Expedited patient-specific assessment of contact stress exposure in the ankle joint following definitive articular fracture reduction. *J. Biomech.* 48 3427–3432. 10.1016/j.jbiomech.2015.05.030 26105660 PMC4636344

[B30] Kohnlein W. Ganz R. Impellizzeri F. M. Leunig M. (2009). Acetabular morphology: implications for joint-preserving surgery. *Clin. Orthop. Relat. Res.* 467 682–691. 10.1007/s11999-008-0682-9 19130159 PMC2635447

[B31] Li G. Sakamoto M. Chao E. Y. S. (1997). A comparison of different methods in predicting static pressure distribution in articulating joints. *J. Biomech.* 30 635–638. 10.1016/s0021-9290(97)00009-2 9165398

[B32] Mavcic B. Pompe B. Antolic V. Daniel M. Iglic A. Kralj-Iglic V. (2002). Mathematical estimation of stress distribution in normal and dysplastic human hips. *J. Orthop. Res.* 20 1025–1030. 10.1016/s0736-0266(02)00014-1 12382969

[B33] Menschik F. (1997). The hip joint as a conchoid shape. *J. Biomech.* 30 971–973. 10.1016/s0021-9290(97)00051-1 9302622

[B34] Murphy L. B. Helmick C. G. Schwartz T. A. Renner J. B. Tudor G. Koch G. G. (2010). One in four people may develop symptomatic hip osteoarthritis in his or her lifetime. *Osteoarthr. Cartil.* 18 1372–1379. 10.1016/j.joca.2010.08.005 20713163 PMC2998063

[B35] Reijman M. Hazes J. M. W. Pols H. A. P. Koes B. W. Bierma-Zeinstra S. M. A. (2005). Acetabular dysplasia predicts incident osteoarthritis of the hip the rotterdam study. *Arthr. Rheum.* 52 787–793. 10.1002/art.20886 15751071

[B36] Styner M. A. Rajamani K. T. Nolte L. P. Zsemlye G. Szekely G. Taylor C. J. (2003). Evaluation of 3D correspondence methods for model building. *Inform. Proc. Med. Imaging Proc.* 2732 63–75. 10.1007/978-3-540-45087-0_6 15344447

[B37] Viceconti M. Olsen S. Nolte L. P. Burton K. (2005). Extracting clinically relevant data from finite element simulations. *Clin. Biomech.* 20 451–454. 10.1016/j.clinbiomech.2005.01.010 15836931

[B38] Volokh K. Y. Chao E. Y. Armand M. (2007). On foundations of discrete element analysis of contact in diarthrodial joints. *Mol. Cell. Biomech.* 4 67–73. 17937111 PMC2692889

[B39] Volokh K. Y. Yoshida H. Leali A. Fetto J. F. Chao E. Y. S. (2006). Prediction of femoral head collapse in osteonecrosis. *J. Biomech. Eng.* 128 467–470. 10.1115/1.2187050 16706598

[B40] von Eisenhart-Rothe R. Eckstein F. Muller-Gerbl M. Landgraf J. Rock C. Putz R. (1997). Direct comparison of contact areas, contact stress and subchondral mineralization in human hip joint specimens. *Anat. Embryol.* 195 279–288. 10.1007/s004290050047 9084826

[B41] Yoshida H. Faust A. Wilckens J. Kitagawa M. Fetto J. Chao E. Y. S. (2006). Three-dimensional dynamic hip contact area and pressure distribution during activities of daily living. *J. Biomech.* 39 1996–2004. 10.1016/j.jbiomech.2005.06.026 16120442

